# An increase in the biogenic aerosol concentration as a contributing factor to the recent wetting trend in Tibetan Plateau

**DOI:** 10.1038/srep14628

**Published:** 2015-09-28

**Authors:** Keyan Fang, Risto Makkonen, Zhengtang Guo, Yan Zhao, Heikki Seppä

**Affiliations:** 1 Institute of Geography, Key Laboratory of Humid Subtropical Eco-geographical Process (Ministry of Education), College of Geographical Sciences, Fujian Normal University, Fuzhou 350007, China; 2 Key Laboratory of Cenozoic Geology and Environment, Institute of Geology and Geophysics, Chinese Academy of Sciences, Beijing 100029, China; 3 Department of Physics, PO Box 64, 00014 University of Helsinki, Helsinki, Finland; 4 Institute of Geographic Sciences and Natural Resources Research, Chinese Academy of Sciences, Beijing 100101, China; 5 Department of Geosciences and Geography, PO Box 64, 00014 University of Helsinki, Helsinki, Finland

## Abstract

A significant wetting trend since the early 1980s in Tibetan Plateau (TP) is most conspicuous in central and eastern Asia as shown in the instrumental data and the long-term moisture sensitive tree rings. We found that anomalies in the large-scale oceanic and atmospheric circulations do not play a significant role on the wetting trend in TP. Meanwhile, the weak correlation between local temperature and precipitation suggests that the temperature-induced enhancement of the local water cycle cannot fully explain the wetting trend either. This may indicate the presence of nonlinear processes between local temperature and precipitation. We hypothesize that the current warming may enhance the emissions of the biogenic volatile organic compounds (BVOC) that can increase the secondary organic aerosols (SOA), contributing to the precipitation increase. The wetting trend can increase the vegetation cover and cause a positive feedback on the BVOC emissions. Our simulations indicate a significant contribution of increased BVOC emissions to the regional organic aerosol mass and the simulated increase in BVOC emissions is significantly correlated with the wetting trend in TP.

Hydroclimate changes in Tibetan Plateau (TP) directly affect the Asian agro-economic livelihood as it is the source region of the major rivers in Asia (e.g. Indus, Yangtze, and Mekong). More importantly, it can indirectly influence the Asian population by modulating the strength of the Asian summer monsoon. Hydroclimate in TP has received increasing attention in recent decades because of the sharp increase in water demands for agricultural and industrial activities in TP and the surrounding areas^1^,^2^,^3^. To project future hydroclimate variations under various warming scenarios, one needs to understand the mechanisms of the current hydroclimate changes in TP. Instrumental data shows that TP has experienced a wetting trend during the current warming^1^,^4^. This wetting trend is most conspicuous during the past millennium as suggested by tree rings over some parts of TP, such as western TP^5^ and northeastern TP^6^. Such hydroclimate changes may be associated with anomalies in large-scale oceanic and atmospheric patterns, which can modulate the available moisture advection and the convection in TP. Meanwhile, the local water cycle can also play an important role in hydroclimate changes in TP. The current warming trend may enhance the water cycle and lead to an increase in precipitation^2^,^6^.

On the other hand, changes in the atmospheric aerosols can modulate the concentration of cloud condensation nuclei (CCN), affecting precipitation in TP. Largely because of the low population and limited anthropogenic influence, TP represents a region with one of the lowest continental annual mean CCN concentrations of 100–200 cm^−3^, which is a magnitude lower than in the surrounding regions towards East and South of TP^7^. The low baseline concentration of aerosols makes the aerosol-cloud interactions in TP susceptible to changes in transported and locally emitted aerosols and precursors. Similar to the circumpolar areas^8^, the biogenic volatile organic compounds (BVOC) emissions from terrestrial vegetation may prompt the generation of secondary organic aerosols (SOA), causing an increase of the CCN and thus probably a wetting trend in TP^8^,^9^,^10^.

This study investigates the potential influences of the three factors on the recent wetting trend in TP. Firstly, we investigate the spatiotemporal patterns of the hydroclimate trends in recent decades in eastern Asia. Then we address the modern wetting trend by studying its relationships with the large-scale oceanic and atmospheric patterns. We further discuss its relationship with the local water cycle. Finally, the relationships between the wetting trend and the BVOC changes are investigated. A process-based dynamic vegetation model, the Lund-Potsdam-Jena-General Ecosystem Simulator (LPJ-GUESS)^11^,^12^, is employed to simulate the BVOC changes in the past decades, because systematic observations of BVOC changes in TP are not available. This model is driven by the local environmental conditions and can simulate the species- and population-level vegetation structures from landscape to global scale and incorporates the modules for BVOC simulations^13^. The global aerosol-climate model ECHAM5.5-HAM2^14^,^15^ is used to simulate BVOC-aerosol coupling in TP.

## The wetting trend in TP in the modern period

**Figure 1:**
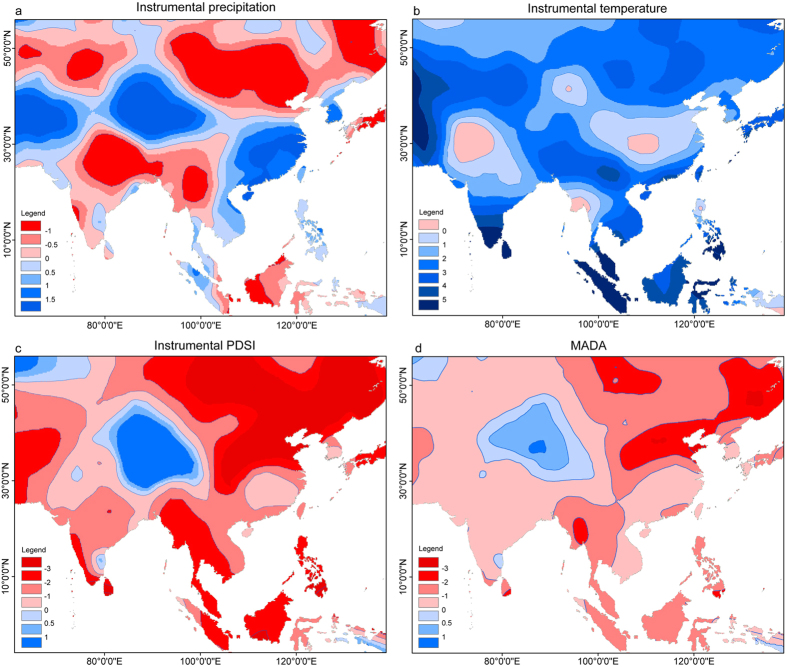
Trends of (a) the instrumental precipitation, (b) the temperature and (c) the Palmer drought severity index (PDSI) from 1951 to 2009 calculated using the Mann-Kendal method, as well as (d) the ratio between mean PDSI from 1981 to 2005 and the reconstructed PDSI from 1300 to 2005 in the monsoonal Asian drought atlas (MADA). The positive values indicate an increasing trend and vice versa. The figure was generated by the software of ArcMap 10.2 and Adobe Illustrator.

The most conspicuous wetting trend in summer since 1951 for central and eastern Asia is observed in TP, particularly in its northern part, in the instrumental precipitation data and Palmer drought severity index (PDSI) (Fig. 1). The increasing trends in instrumental precipitation records are also observed in south central Asia and southern China, but not in the instrumental PDSI data (Fig. 1). This is because the intensified evaporation under the concurrent warming contradicts the increase in precipitation in these regions^16^,^17^. However, the warming in TP may have limited influence on the evaporation due to cold environments. Therefore, the increase in PDSI is most significant in TP. Tree-ring data show that the reconstructed PDSI over TP in the past two decades is the highest over the past 7 centuries (Fig. 1). It should be noted that the long-term trends are not sufficiently reliable in these tree-ring based reconstructions as most of the low-frequency variations were removed^18^. There are other tree-ring based reconstructions that reveal the hydroclimate changes in northeastern TP over the past 3500 years^6^ and in western TP over the past millennium^5^, also suggesting that the current wetting trend in TP is the most conspicuous. It is therefore clear that the wetting trend in TP is most significant over central and eastern Asia and is very likely the most intense period of precipitation increase over the past centuries or millennia.

### Linkages with large-scale climate anomalies and local water cycles

**Figure 2:**
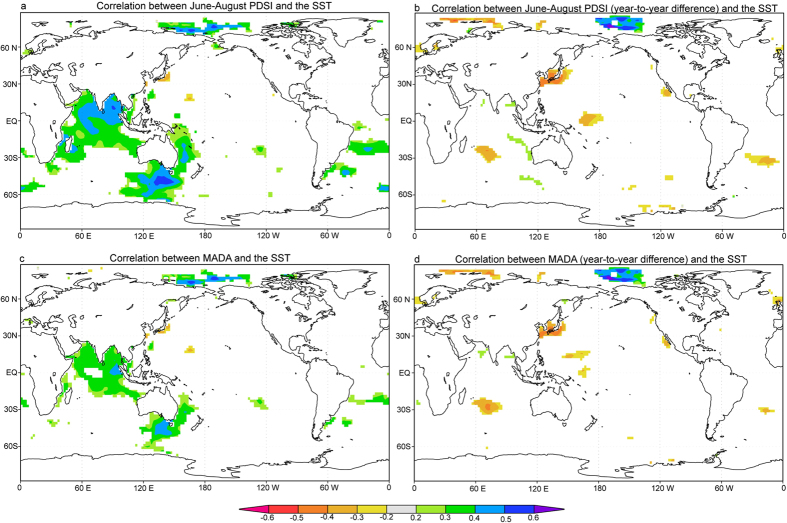
Correlations between the SST from 1951–2005 and (a) the instrumental summer (June-August) PDSI, (b) the instrumental summer PDSI with year-to-year difference, (c) the reconstructed summer PDSI and (d) the reconstructed summer PDSI with year-to-year difference for period 1951–2005. The figure was generated by the KNMI explorer (http://climexp.knmi.nl) and Adobe Illustrator.

A potential factor accounting for the wetting trend in TP is the anomalies in the large-scale oceanic and atmospheric patterns. As shown in Fig. 2, both the instrumental and reconstructed PDSI show significant correlations with sea surface temperature (SST) of the northern Indian Ocean, Arctic Ocean and Southern Ocean, consistent with previous studies^6^,^19^,^20^. We further calculated the correlations with SST using the year-to-year difference data to investigate the linkages at the interannual timescale. Significant correlations with SST in the northern Indian Ocean, Arctic Ocean and Southern Ocean are absent at the interannual timescale, suggesting that the linkages with SST in these areas are largely caused by their interdecadal variations^6^.

However, it is still not clear whether these interdecadal teleconnections are robust through time due to the shortness of the instrumental data spanning only a few decades. We thus analyzed the relationships between the interdecadal changes of hydroclimate in TP and the oceanic and atmospheric patterns using the reconstructed PDSI in TP and the reconstructed SST. We generated the mean PDSI values from 15 grids in TP where wetting trends are observed in the instrumental precipitation and PDSI data, as well as the reconstructed PDSI as revealed in Fig. 1. A 51-year Gaussian low-passed filter was then applied to highlight interdecadal climate changes (Figure S2). For the past 7 centuries, high correlations between interdecadal hydroclimate changes in TP and SST in the northern Indian Ocean observed in the modern period are absent, indicating that the linkages to SST in the northern Indian Ocean are not robust through time (Figure S3). The warming of the northern Indian Ocean can increase the content of water vapor in air, and the water vapor carried by the Asian summer monsoon can influence some of the surrounding regions of TP, such as the low-lying valleys, but can seldom penetrate to the interiors of TP due to its high elevation. In addition, it is not likely that the wetting trend in TP can be related to the strength of the Asian summer monsoon that has weakened since the 1980s (Figure S4). The weakening of the Asian summer monsoon can explain the drying trend in the southern rim of TP, i.e. the Himalayan Mountain regions, as its precipitation is governed by the Asian summer monsoon. Thus the wetting trend in TP is particularly strong in its northern part. Relatively high correlations are observed over the Southern Ocean for the past 7 centuries. The Southern Ocean has been widely documented to modulate the strength of the Asian summer monsoon^21^, while the physical linkages between SST in Southern Ocean and the hydroclimate changes in TP is unclear. In conclusion, it is not likely that the large-scale oceanic and atmospheric anomalies would cause the wetting trend in TP.

**Figure 3:**
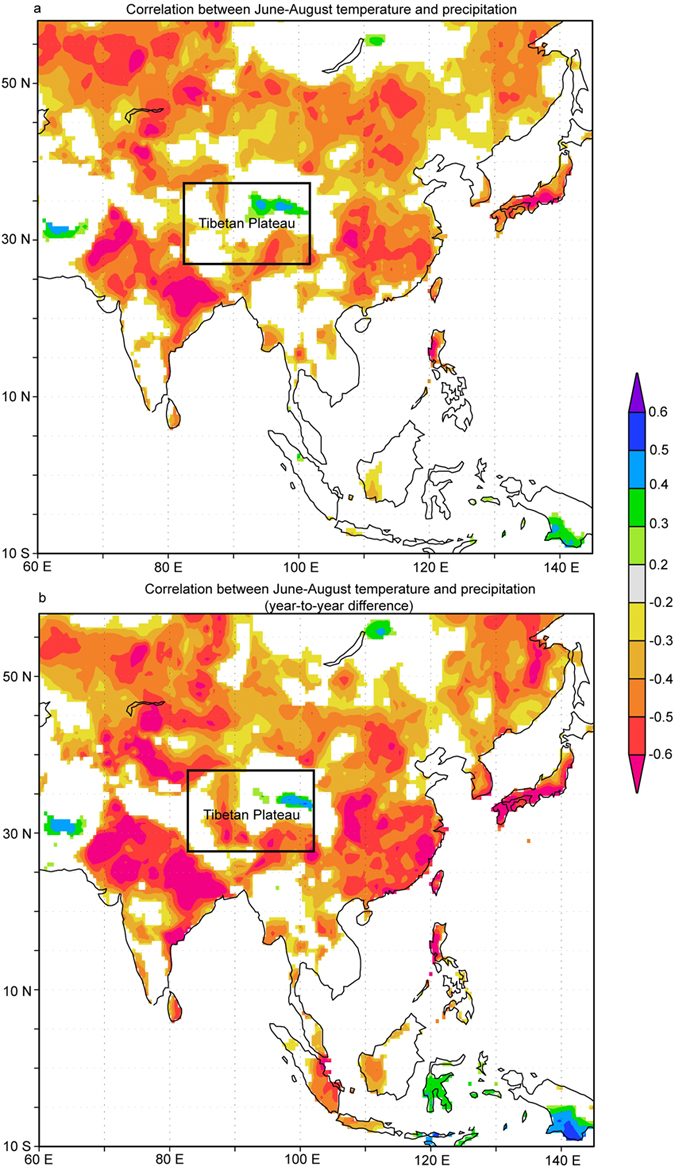
(**a**) Correlations between summer (June-August) temperature and precipitation from the CRU TS 3.1 dataset during the modern period since 1951 and (**b**) correlations between summer temperature and precipitation for the year-to-year difference data. Insignificant (p > 0.1) correlations are left blank. This figure was produced using the KNMI explorer (http://climexp.knmi.nl) and Adobe Illustrator.

Apart from the large-scale oceanic and atmospheric patterns, changes in the local water cycle may also influence the hydroclimate in TP^6^,^22^. The warming can increase the moisture content in air by enhancing the water cycle and elevating the atmospheric water retention. If so, there should be strong correlation between local temperature and precipitation. However, we did not find significant correlation between temperature and precipitation in TP (Fig. 3a). Even negative correlations are found between the precipitation and temperature for some regions in TP at the interannual timescale (Fig. 3b). Although there are similarities between a precipitation reconstruction from northeastern TP and temperature reconstruction at interdecadal timescales, considerable mismatches are also observed^6^. We therefore state that the local water cycle cannot fully explain the wetting trend in TP. The low linear correlations between local temperature and precipitation, particularly at the interannual timescale, suggest the presence of possible nonlinear relationships between local temperature and precipitation that are modulated by other factors.

### Linkages with the BVOC emissions

One remaining factor that may contribute to the wetting trend is the changes in BVOC and the associated CCN concentrations. Prior to the BVOC simulations, we first evaluated the efficiency of the LPJ-GUESS to simulate the distribution of vegetation by comparing the modeled and natural plant functional types (PFTs) derived from the ISLSCP II potential vegetation cover dataset based on the satellite data^23^. Northern TP with sparsely distributed grass is expressed as desert in the satellite data and as herbaceous vegetation in the simulations since the current model does not contain the PFT of desert. The simulated herbaceous vegetation does not expand to some parts of the southern TP, which is likely due to biases in the gridded climate data that drive the model. The climate data are the interpolated data from the meteorological data that may be largely influenced by the data from the low-lying stations in the southern rim of TP and thus overestimate the local precipitation and temperature. In general, the LPJ-GUESS can reproduce the patterns of the real vegetation distribution (Figure S5 and Table S2), validating our use of this model to simulate the BVOC changes.

**Figure 4:**
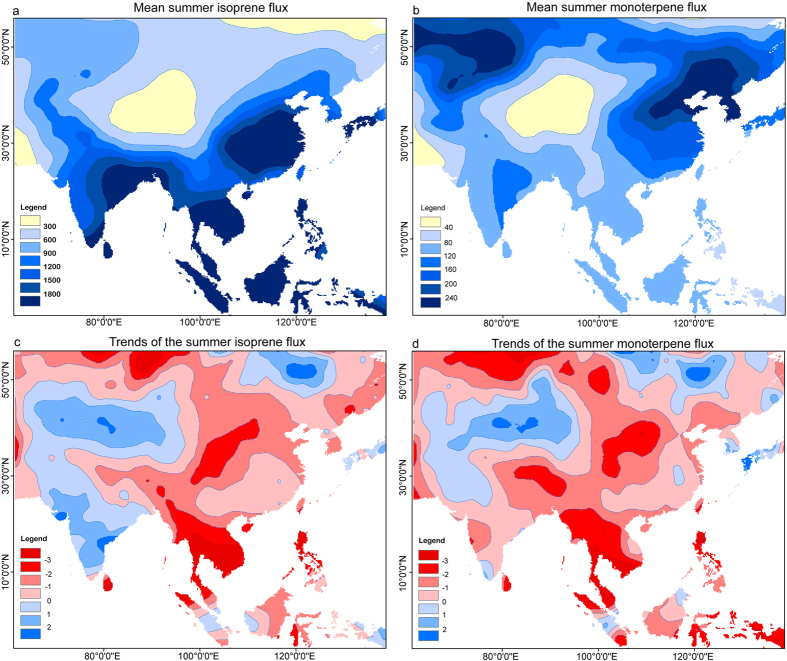
Maps of (a) the monthly mean of the summer (June-August) isoprene flux (ug), (b) the monthly mean of the summer monoterpene (ug), (c) the trends of the monthly mean of the summer isoprene and (d) the monthly mean of the summer monoterpene in the simulations of the modern period from 1950 to 2005. This figure was generated using the software of ArcMap 10.2 and Adobe Illustrator.

Both the isoprene and monoterpene emissions are the lowest in TP (Fig. 4a,b), consistently with the previous findings^7^, which is related to the low biomass and cold temperature. The area with the lowest BVOC emissions focus on northern TP and surroundings. This is because the simulation overestimates the biomass over the southern rim of TP as aforementioned. Our results indicate that the highest summer isoprene emissions occur over southern and southeastern Asia (Fig. 4a). The highest summer monoterpene emissions are found in the central and eastern High Asia (Fig. 4b), although the annual monoterpene emissions are higher in the low latitudes^13^. This is because the gradients of temperature and radiation in summer is less strong and the monoterpene emissions are more depend on the PFT related emission capacities^10^,^13^ than on the environmental gradients.

**Figure 5:**
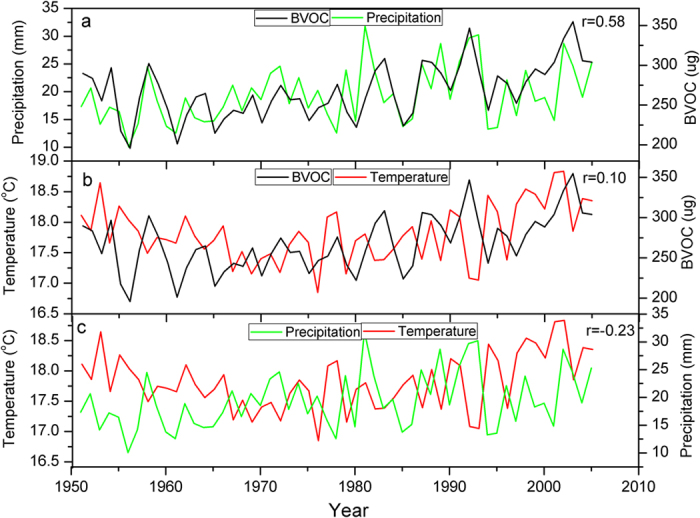
Comparisons between the simulated monthly mean of the summer (June-July-August) BVOC emissions and the (a) monthly mean of the summer precipitation (r = 0.58), the (b) monthly mean of the summer temperature (r = 0.10) and the (c) monthly mean of the summer precipitation and temperature (r = −0.23) since 1950 in Tibetan Plateau.

**Figure 6:**
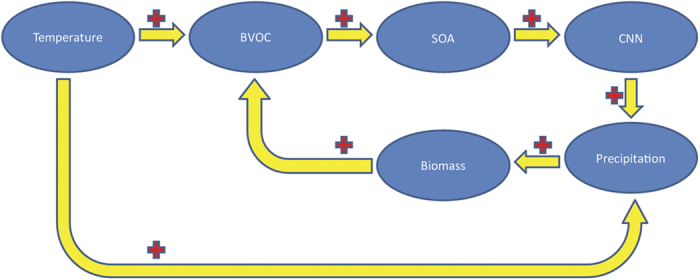
Feedback loops between temperature, biogenic volatile organic compound (BVOC), secondary organic aerosol (SOA), cloud condensation nuclei (CCN), precipitation and biomass, as a suggested model for the recent wetting trend in TP. The crosses indicate the positive feedback effects.

Increasing trends are observed in TP for both the isoprene and monoterpene emissions and in the Indian subcontinent for the isoprene (Fig. 4c,d). Similar to the northward shift of the region with the lowest simulated BVOC emissions in TP, the region with an increasing trend in BVOC emissions in TP also shifts northward due to the overestimation of the biomass in its southern part. As shown in Fig. 5, the BVOC emissions in TP are significantly correlated with local precipitation (r = 0.58), while the correlations between BVOC emissions and temperature and between temperature and precipitation are insignificant. The high correlation between precipitation and BVOC emission suggests a strong linkage of BVOC on precipitation. The BVOC emissions in the current model is a function of photosynthesis and climate^13^. The warming trend can be associated with a reduction in photosynthesis due to a partial stomatal closure in response to a climatic drought stress, which may contradict the warming induced increase in BVOC emissions at the interannual timescale. Thus it is insignificant for the linear correlations between temperature and BVOC emissions that are largely influenced by the interannual variations. However, the rising trend of temperature, BVOC emissions and precipitation at the interdecadal timescale suggest the linkages between them at the interdecadal timescale. Instead of direct influences of temperature on BVOC emissions, we hypothesize that warming and associated enhanced water cycle in TP can cause increasing trend in biomass and emissions of BVOC, a source of SOA and CCN^13^. Via positive feedback loops, the increase in CCN can increase the precipitation and thus increase the biomass in the dry TP, which can in turn cause an increase of the BVOC (Fig. 6). These feedback loops can cause the nonlinear relationships between temperature and precipitation and can explain the low linear correlations between them. The positive feedback between temperature and precipitation is supported by the warming trend and the wetting trend in recent decades. An increase in vegetation cover since the 1980s is observed in TP as revealed in the remote sensing data^24^. An increase in BVOC emission can play a particularly important role in regions with low baseline BVOC such as TP.

The main uncertainties in the proposed feedback loop include the processes in linking the increased BVOC emissions and the increase in the CCN production. We thus employed the ECHAM5.5-HAM2 model to study the interactions between BVOC emissions, aerosol formations and climate in TP. An increase by 20% of the BVOC emissions since 1950 is observed for northern TP and surroundings, where an increase in BVOC is most conspicuous. Accordingly, we conducted the controlled simulation based on the present-day BVOC emissions and the perturbed TP simulation assuming an increase of 20% in BVOC emission in northern TP and surroundings. A relative increase of 5–10% in simulated summer (June-August) organic aerosol mass is observed in the region (Figures S6 and S7). The SOA from BVOC emissions is condensing primarily to the Accumulation mode with larger aerosol size (100 < d_p_ < 1000 nm) than that of Aitken-mode with smaller aerosol size (10 < d_p_ < 100 nm) (Figure S8). However, the model indicates a low sensitivity of simulated CCN concentration to the increased BVOC emissions. This is largely because simulating the CCN-modifying effects of SOA formation require the information on aerosol distribution in TP to constrain model, which is insufficient due to scarcity of observations. To further validate the feedbacks loops, we suggest future monitoring and experimental studies on the BVOC emissions in Tibetan Plateau and the use of aerosol-climate models with higher horizontal resolution and more detailed chemistry and aerosol data. Nevertheless, the model indicates both low baseline aerosol concentrations over TP and a significant signal from BVOC emission to overall organic aerosol concentrations.

In summary, this study highlights that a strong summer wetting trend has occurred in TP since the 1980s, which is very likely the most conspicuous wetting during the past centuries or even millennia. The wetting trend in TP is not likely to be caused by the anomalies in the oceanic and atmospheric patterns. Enhanced local water cycle may contribute to the wetting trend. However, the linear correlation between local temperature and precipitation is not strong. The increase in the BVOC emissions can lead to an increase the SOA and CCN and hence enhance the wetting trend, which can in turn increase the vegetation cover and further accelerates the BVOC emissions. The positive feedbacks among temperature, BVOC emissions, precipitation and vegetation cover can thus lead to nonlinear relationships between temperature and precipitation, resulting in a low linear correlation between local temperature and precipitation. These feedback loops are supported by the high correlation between local precipitation and the BVOC emissions simulated by LPJ-GUESS. Further aerosol-climate modeling also indicates an increase in organic aerosol mass due to the increase in BVOC emissions in TP.

## Methods

### The climate data

We employed the precipitation and temperature data from the 2.5° × 2.5° gridded CRU TS3.1 dataset^25^. The PDSI from a global dataset with a 2.5° × 2.5° resolution^16^ was also used to study the moisture variability. Since our focus is on the summer (June-July-August) PDSI in TP, the limited ability of PDSI in modeling the snow conditions can be negligible^18^. We only used the relatively reliable portion of these instrumental records since 1951 when most of the meteorological stations were established^18^. Apart from the instrumental records, we also employed the MADA since 1300 that is consist of 534 reconstructed summer (June-July-August) PDSI grids^18^. The local features of the moisture variability were reconstructed using the search radius to locate the nearby tree-ring chronologies in the point-by-point regression method. Sufficient number of tree-ring chronologies were available in and near TP, which validates the reconstruction variability of MADA. We investigated the relationships between the long-term hydroclimate teleconnection and the reconstructed SST derived from an annual temperature reconstruction of 2592 grids with a spatial resolution of 5° × 5° across the entire globe spanning from 500–2006^26^. The proxy data in this reconstruction are composed of 1138 series from 9 different proxies with most of them starting from 1600s. The MADA and the reconstructed SST are relatively independent because that a small fraction of the proxy records are shared in both datasets.

### Statistical and modeling methods

Trends in the climate records were estimated using the rank-based distribution-free Mann-Kendall method^27^,^28^. This non-parametric method is widely used because it is simple, robust, can cope with the extreme and missing values and requires no assumptions for a specific distribution. However, the detrending procedures used in the MADA removed much of the long-term trends. We therefore did not apply the Mann-Kendall method to evaluate whether the modern wetting trend is most intense in the reconstruction period. Instead, we calculated the difference between the mean PDSI in modern period (i.e. from 1981 to 2005) and the mean PDSI of the entire period to evaluate the intensity of current wetting trend.

Our LPJ-GUESS simulation was driven by the monthly temperature and precipitation since 1951 from the CRU TS 3.1 dataset (Appendix). The soil texture data were in the form of a soil code following the BIOME model^29^,^30^. The model spinup was implemented by running the model for 500 years by repeatedly using the first 50 years of the climate data. The current model does not take into account the interactions between neighboring regions (e.g. the water and seeds exchanges) and simulates each grid cell independently. The model simulates the daily-level processes of physiological activities including the photosynthesis and respiration, as well as the heat, carbon, BVOC and water exchanges between the vegetation, the soil and the atmosphere^11^,^12^,^13^. The current version of LPJ-GUESS generates the two major sources of BVOC, i.e. the isoprene and monoterpene^8^,^10^,^13^. The BVOC simulations are modulated by the emission capacities of isoprene and monoterpene as shown in Table S1. Monoterpene and isoprene in the aerosol-climate simulations using the ECHAM5.5-HAM are considered as precursors for the SOA. A SOA yield of 15% for monoterpene and 5% for isoprene is assumed. The model is integrated over one year after 3-month spinup period.

## Additional Information

**How to cite this article**: Fang, K. *et al.* An increase in the biogenic aerosol concentration as a contributing factor to the recent wetting trend in Tibetan Plateau. *Sci. Rep.* **5**, 14628; doi: 10.1038/srep14628 (2015).

## Supplementary Material

Supplementary Information
