# Supplementary material for: An increase in the biogenic aerosol concentration as a contributing factor to the recent wetting trend in Tibetan Plateau
Source: Sci Rep. 2015 Sep 28;5:14628. doi: 10.1038/srep14628 (PMC4585965; doi:10.1038/srep14628)
Supplement: Supplementary Information [file srep14628-s1.doc]

**Appendix**

**for**

**An increase in the biogenic aerosol concentration as a contributing factor to the recent wetting trend on Tibetan Plateau**

Keyan Fang^1,2*^, Risto Makkonen^3^, Zhengtang Guo^2^, Yan Zhao^4^, Heikki Seppä^5^

1. Key Laboratory of Humid Subtropical Eco-geographical Process (Ministry of Education), College of Geographical Sciences, Fujian Normal University, Fuzhou 350007, China

2. Key Laboratory of Cenozoic Geology and Environment, Institute of Geology and Geophysics, Chinese Academy of Sciences, Beijing 100029, China

3. Department of Physics, PO Box 64, 00014 University of Helsinki, Helsinki, Finland

4. Institute of Geographic Sciences and Natural Resources Research, Chinese Academy of Sciences, Beijing 100101, China

5. Department of Geosciences and Geography, PO Box 64, 00014 University of Helsinki, Helsinki, Finland

^*^ To whom correspondence should be addressed:

[kujanfang@gmail.com](mailto:kujanfang@gmail.com)

**The Study region**

TP is the world's "roof" of 2,500,000 km^2^ with an average elevation over 4500m. The elevation increases sharply from the southern rim of the TP and decreases gradually towards northern TP (Figure S1a). The low-lying area in northeastern TP is the Qaidam basin, which is dominated by deserts. Generally, TP is characterized by a highland continental climate with long, dry winter and the vegetation is mainly composed by grasslands, shrubs and tundra (Figure S1b). Asian summer monsoon penetrates into some valleys, particularly in southeastern TP, leading to a warm and humid local climate. In general, the precipitation decreases from the southern and eastern TP towards the northern and western TP. The peak summer precipitation in eastern Asia is observed between the Bay of Bengal and the southern rim of TP (Figure S1c). Forests are present in the southern rim of TP and in some valleys of eastern TP. The summer temperature in TP is the lowest in eastern Asia due to its high elevation (Figure S1d).

**The LPJ-GUESS and ECHAM5.5-HAM2**

The LPJ-GUESS is an improved version of the LPJ-Dynamic Global Vegetation Model (LPJ-DGVM), which employs replicated "patches" (herein 15) to represent different types of dynamics of individuals or cohorts in a grid cell. The size of the patches is approximately the same as the maximum area impacted by a large individual. The model employs the plant functional type (PFT) to represent the structure and function of various plants species of a particular patch in order to simplify the data to facilitate simulations with the model. This study uses the 11 standard PFTs that have been widely used in LPJ-GUESS simulations as shown in Table S1. In LPJ-GUESS, the vegetation is driven by the local environmental conditions, including temperature, precipitation, soil, carbon dioxide and sunshine. Thus the LPJ-GUESS can simulate the different vegetation biomes in this vast area with different environmental conditions. Bioclimatic factors that determine the climatic boundaries of tree species include the minimum growing degree-day sum over 5°C, minimum and maximum temperatures of the coldest month, and minimum temperature of the warmest month. On an annual timestep, LPJ-GUESS simulates the plant growth and its allocations to leaf, sapwood and fine roots, as well as the stochastic processes of population dynamics, mortality and natural disturbances (e.g. fire). Establishments of new cohorts are controlled by the establishment rate and the potential productivity and the mortality rate is determined by stress, senescence and fire disturbances in the model. The BVOC emission herein is simulated via the BVOC module that was added to the trunk version of the LPJ-GUESS model. For BVOC simulations, it assigns a fraction of the electron flux generated for photosynthesis to BVOC formation. The simulated BVOC emission is a function of plant photosynthesis and climate. The BVOC module initializes the fraction of standard emission rates to support the emission capacities for a certain species. The emission capacities are emissions standardized to 30°C and 1000 μmol PAR m^-2^ s^-1^.

The ECHAM5.5-HAM2 incorporates the features of the version 5.5 of the atmospheric general circulation model ECHAM5 [^1^](#_ENREF_1) and the aerosol model of HAM2 [^2^](#_ENREF_2). The model can not only simulate the chemical and physical processes of the aerosols at micro- and global scales but also predict direct and indirect radiative feedbacks of aerosols to climate. The aerosol module HAM includes the most climate relevant aerosol components sulfate, black carbon, organic carbon, dust and sea salt. The aerosol microphysics in the model is represented by seven log-normal modes in the module M7 [^3^](#_ENREF_3), covering nucleation, Aitken, accumulation and coarse modes. The simulations are nudged against ERA-INTERIM reanalysis for year 2000 in order to minimize model variability due to changes in meteorology. Model results are analyzed against changes in aerosol mass and number concentrations.

**
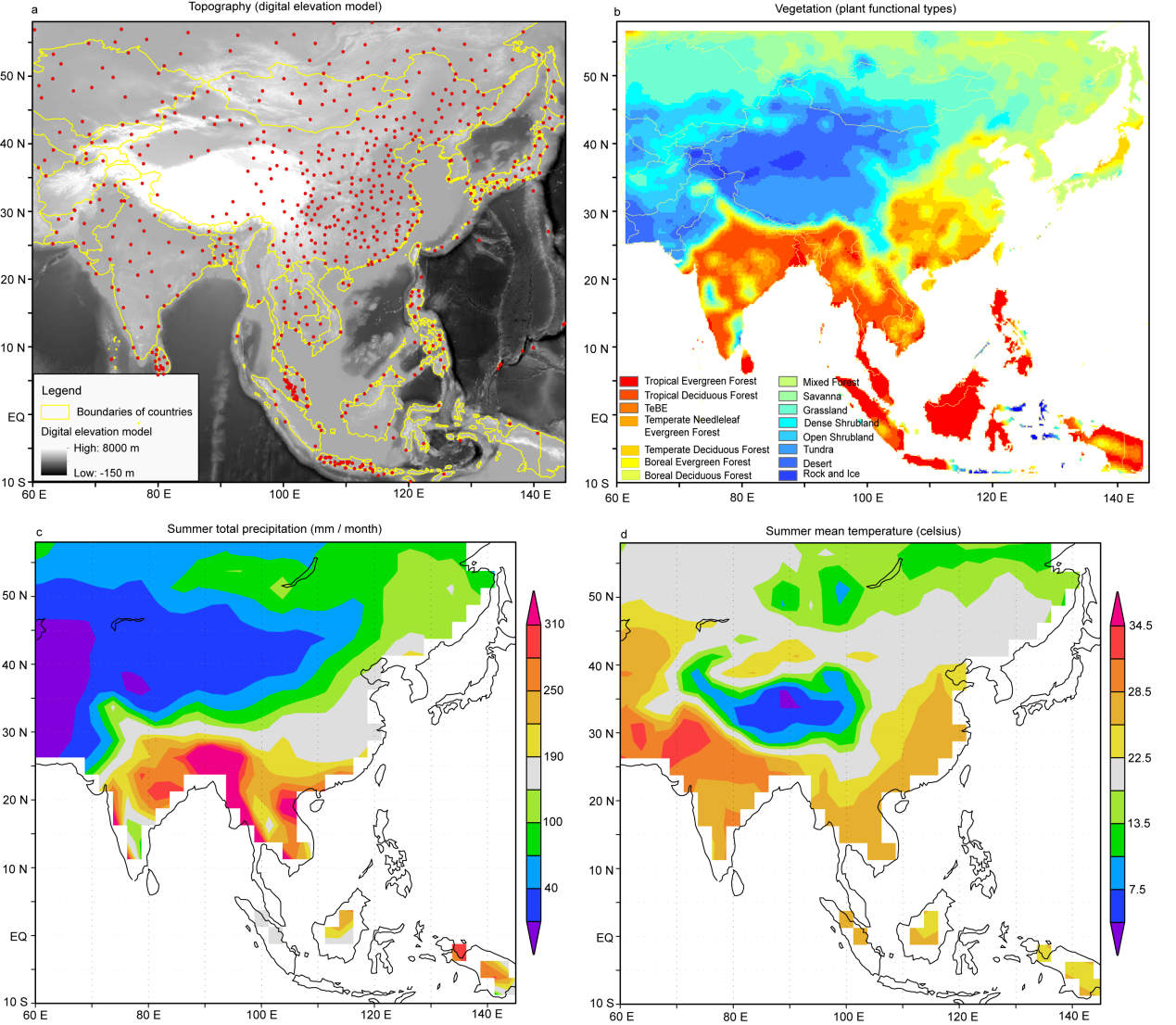
**

**Figure S1**. Map of (a) the topography (digital elevation model) and the locations of the major meteorological stations (red dots) for the entire region (621 stations) and Tibetan Plateau (26 stations) derived from a dataset of 532 stations of the globe (http://cdiac.ornl.gov/ftp/tr051/station.inv), (b) the major plant functional types derived from the ISLSCP II potential vegetation cover dataset based on the satellite data [^4^](#_ENREF_4) (The abbreviations of the plant functional type (PFT) as the same as in Table S1), (c) the mean summer (June-August) precipitation (mm / month) and (d) the summer mean temperature ((°C)) derived from the CRU TS 3.1 dataset for the period 1951-2009. This figure was produced using the ArcMap 10.2 and Adobe Illustrator software.


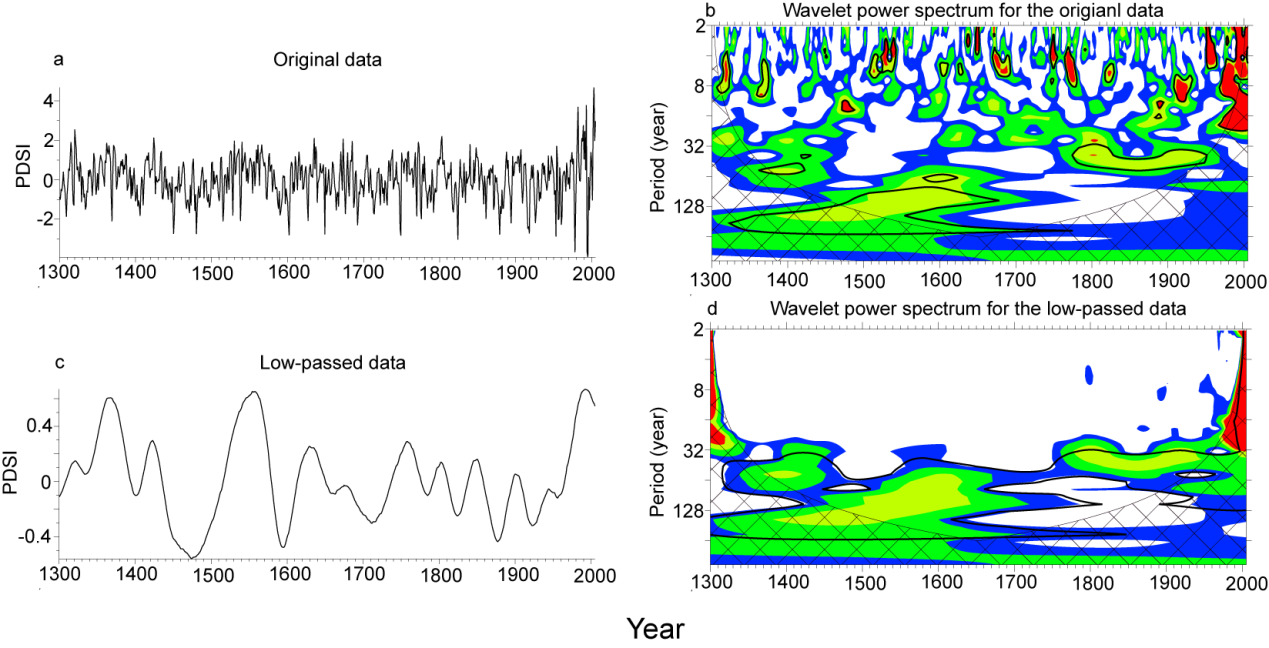


**Figure S2**. (a) The mean reconstructed PDSI in TP in MADA, (b) the wavelet the reconstructed PDSI, (c) the low-passed (51-point Gaussian filter) mean reconstructed PDSI in TP in MADA and (d) its wavelet spectra.


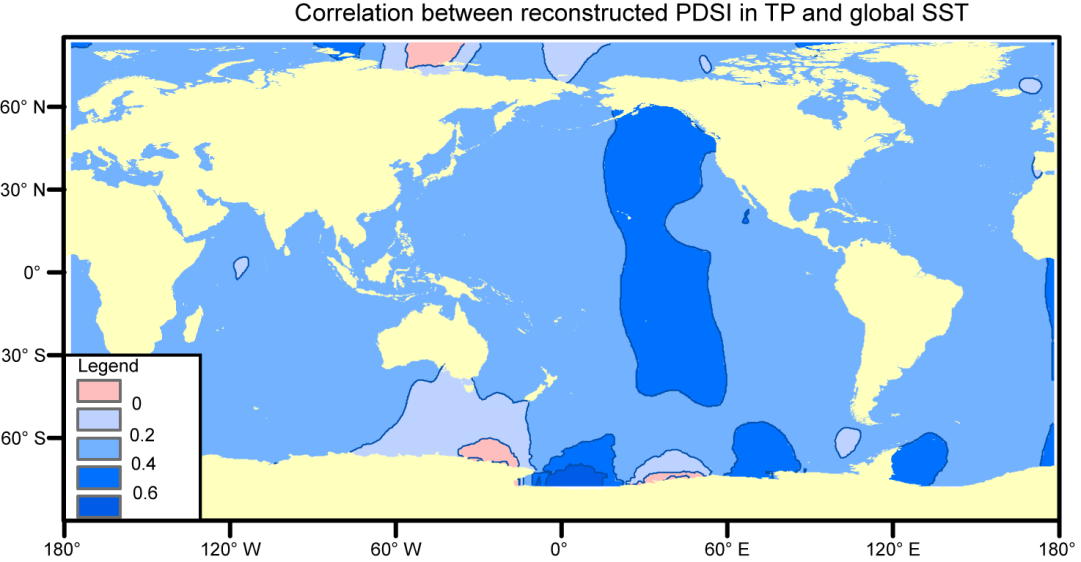


**Figure S3**. The correlations of the low-passed (51-point Gaussian filter) mean reconstructed PDSI in TP with the low-passed reconstructed global SST over their common period 1300-2005. The figure was generated from the ArcMap 10.2 and Adobe Illustrator software.


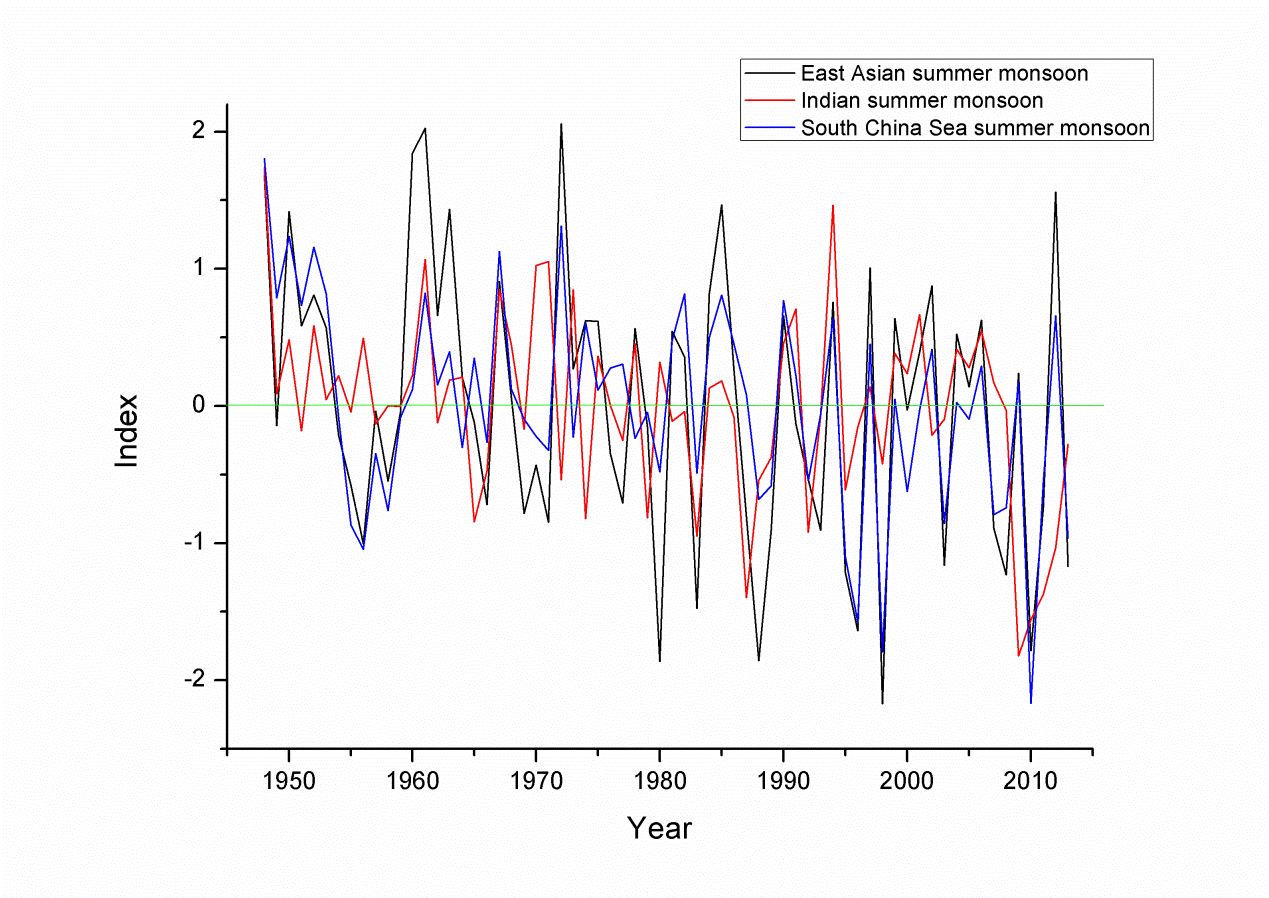


**Figure S4**. Dynamics of the Asian summer monsoon index since 1948 derived from the pressures at 850 hpa [^5^](#_ENREF_5).


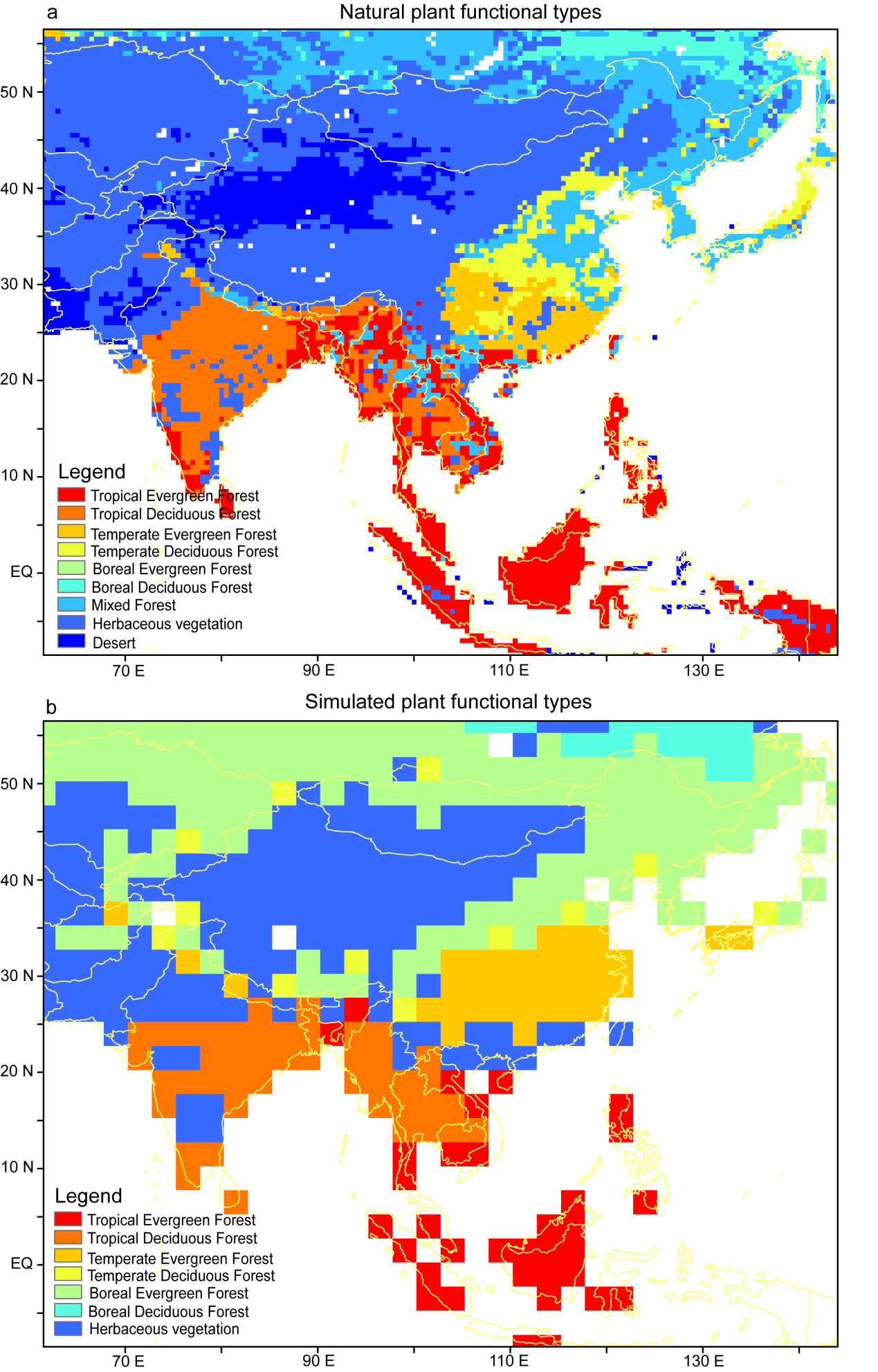


**Figure S5**. (a) The natural plant functional types (PFT) derived from the ISLSCP II PNV Cover dataset that is based on the satellite data and (b) the simulated dominant PFT the maximum leaf area index in each grid for the period in the LPJ-GUESS simulations. Note that the PFT shown herein are the re-classified PFT according to the PFT definitions in the original datasets of the observational and simulated vegetation as shown in Table S2. For example, the herbaceous vegetation are based on the satellite-based observations, which refers to either the C3 or the C4 grasses in the simulations. The figure was generated from the software of the ArcMap 10.2 and Adobe Illustrator.

**
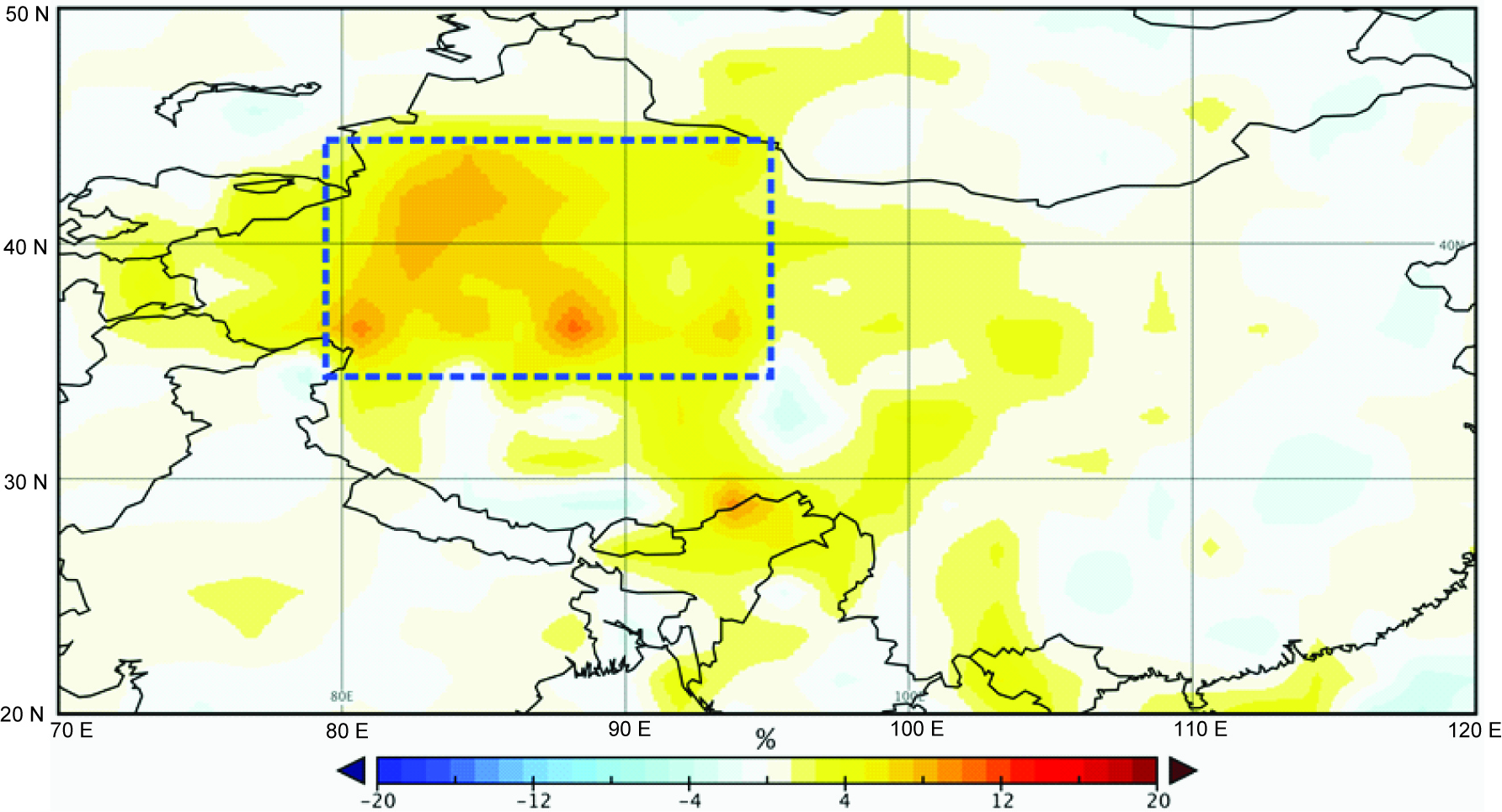
Figure S6**. Relative summer (JJA) increase in organic aerosol mass due to 20% increase in BVOC emissions over TP region (blue rectangle). Aerosol mass is averaged over three lowest model layers. This figure was produced using the PanoplyWin software.

*
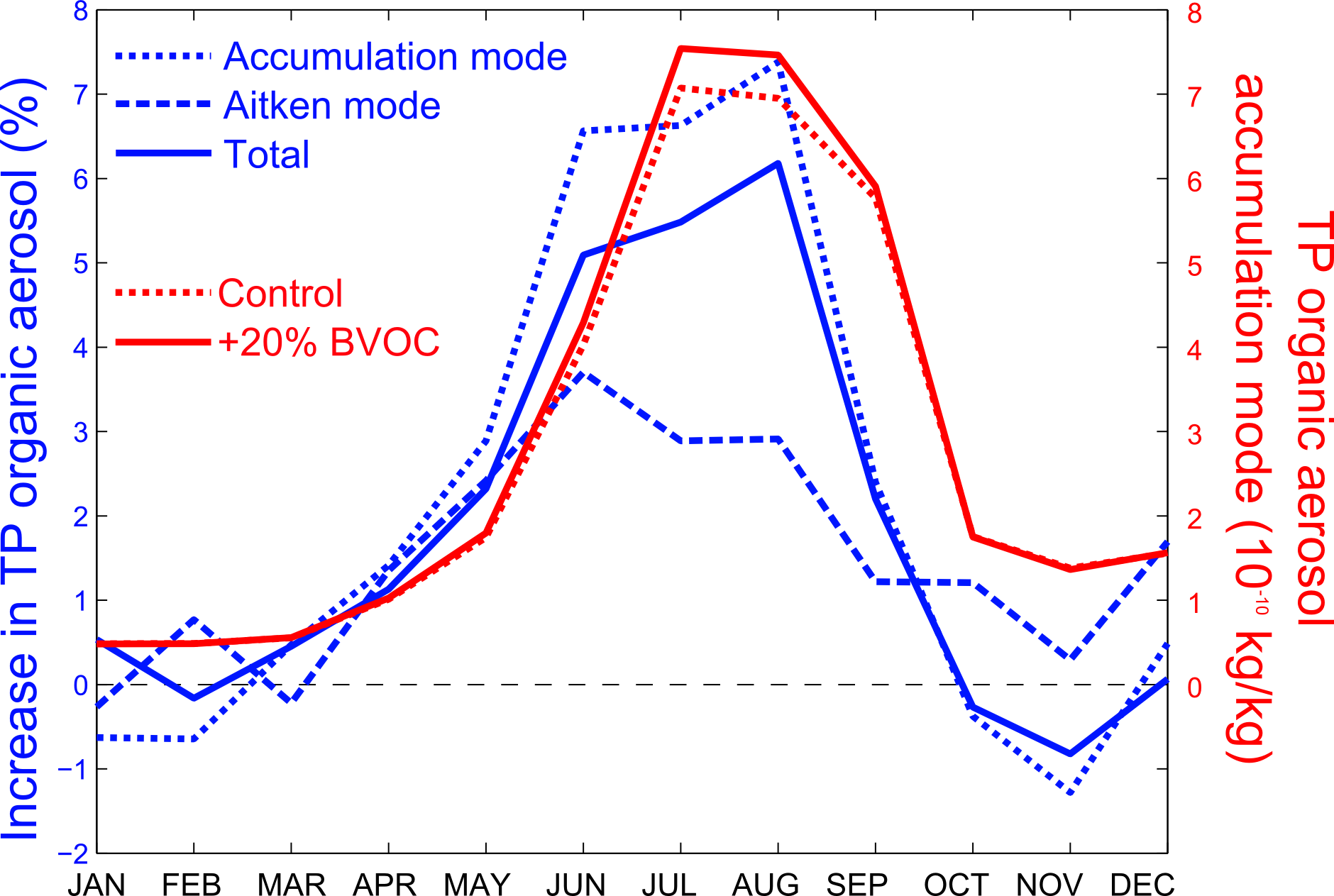
*

**Figure S7**. Increase in TP organic aerosol mass (%, blue lines) in Aitken-mode (dashed) and accumulation mode (dotted), as well as total organic aerosol mass (solid). Also shown is the TP area-averaged organic aerosol mass mixing ratio in accumulation mode (red) for control simulation (dotted) and an increase of 20% BVOC simulation (solid). The size ranges of the aerosol particles in Aitken mode (10<d_p_<100 nm) and Accumulation mode (100<d_p_<1000 nm) are different.

*
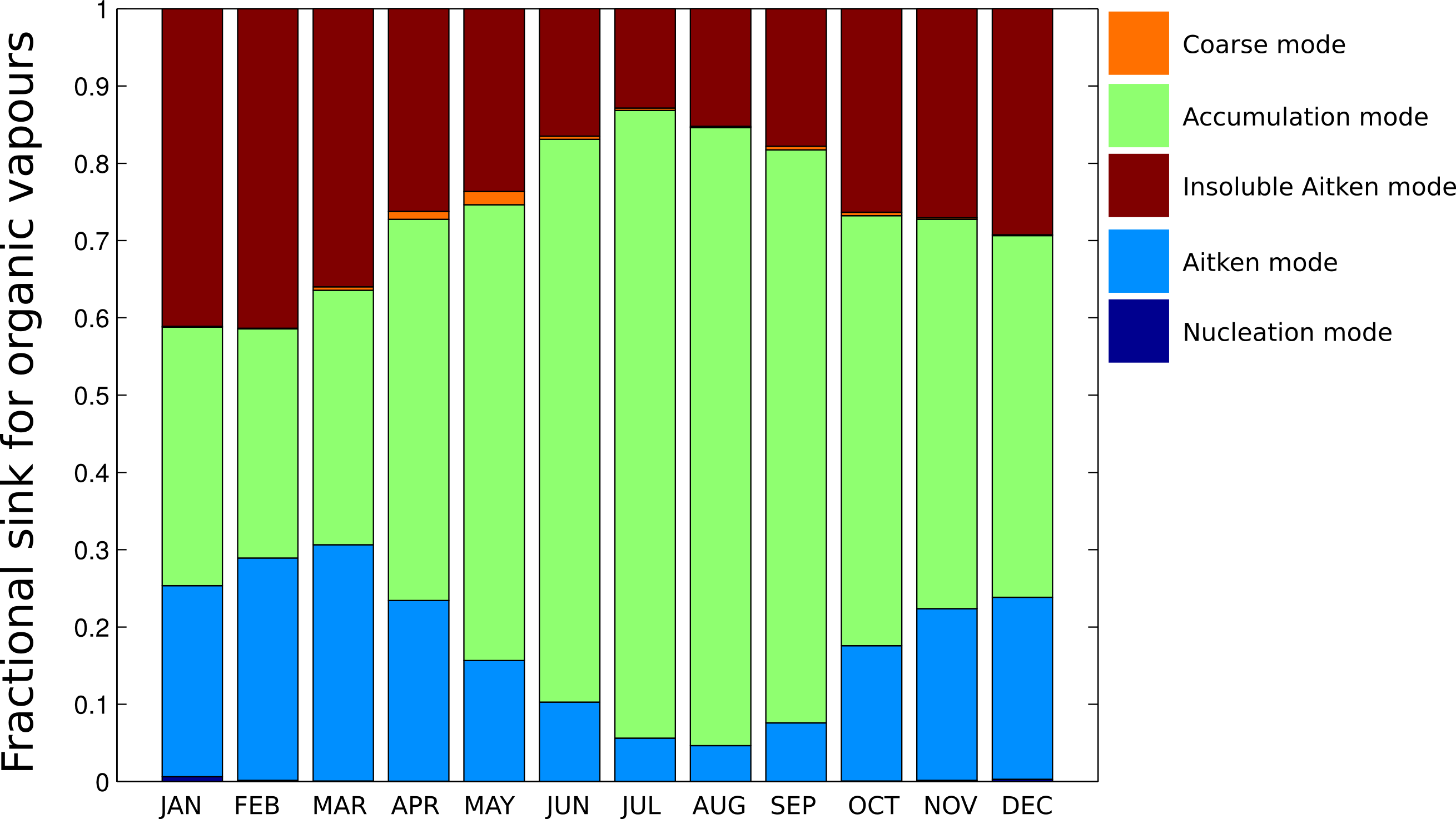
***Figure S8**. Fractional sink contribution from ECHAM-HAM aerosol modes for condensing organics, averaged over Tibetan Plateau derived from a controlled simulation with 20% increase in biogenic volatile organic compounds (BVOC). During summer, even 80% of organics are condensing to Accumulation mode. The size ranges are distinct for the Coarse mode (d_p_ >1000 nm), Accumulation mode (100<d_p_<1000 nm), Aitken mode (10<d_p_<100 nm) and and Nucleation mode (d_p_<10 nm).

**Table S1.** Parameters of the 11 plant functional types (PFT).

| Species/PFT | Shade  tolerance | GDD_5_ | Tc_min | Tc_max | Tw_min | eps_iso | eps_mon |
| --- | --- | --- | --- | --- | --- | --- | --- |
| TrBE | yes | none | 15.5 | none | none | 24 | 0.8 |
| TrIBE | no | none | 15.5 | none | none | 24 | 0.8 |
| TrBR | no | none | 15.5 | none | none | 45 | 2.4 |
| BNE | yes | 500 | -30 | -1 | 5 | 8 | 4.8 |
| BINE | no | 500 | -30 | -1 | 5 | 8 | 4.8 |
| BNS | no | 500 | none | -2 | none | 8 | 4.8 |
| TeBS | yes | 1100 | -14 | 6 | 5 | 45 | 1.6 |
| IBS | no | 350 | -30 | 7 | none | 45 | 1.6 |
| TeBE | yes | 2000 | 0 | 10 | 5 | 24 | 1.6 |
| C3 | none | none | none | none | 0 | 16 | 1.6 |
| C4 | none | none | 15.5 | none | none | 8 | 4 |

**^*^**TrBE: Tropical broadleaved evergreen tree; TrIBE: Tropical broadleaved evergreen tree (intolerant to shade); TrBR: Tropical broadleaved raingreen tree; BNE: Boreal needleleaved evergreen tree (shade tolerant); BINE: Boreal needleleaved evergreen tree (shade intolerant); BNS: boreal needle-leaved summer-green tree; TeBS: shade-tolerant temperate broadleaved summer-green tree; IBS: shade-intolerant broadleaved summer-green tree; TeBE: Temperate broadleaved evergreen tree; C3: C3 grass; C4: C4 grass. GDD_5_: minimum annual sum of daily temperatures above 5°C; Tc_min (Tc_max): minimum (maximum) coldest month mean temperature allowable for establishment (°C); Tw_min: minimum warmest month mean temperature for establishment; eps_iso: isoprene emission capacity (ug Cg^-1^ h^-1^); eps_mon: monoterpene emission capacity (ug Cg^-1^ h^-1^).

**Table S2** The classification of vegetation groups based on the plant functional type (PFT) in ISLSCP II PNV dataset and the simulated PFT in LPJ-GUESS. The abbreviations of the PFT are the same as Table S1.

| **Classified vegetation** | **PFT in LPJ-GUESS** | **PFT in the ISLSCP II PNV Cover dataset** |
| --- | --- | --- |
| Tropical evergreen forest | TrBE; TrIBE | Tropical evergreen forest |
| Tropical deciduous forest | TrBR | Tropical deciduous forest |
| Temperate evergreen forest | TeBE | TeBE; Temperate needleleaf evergreen Forest |
| Temperate deciduous forest | TeBS; IBS | Temperate deciduous forest |
| Boreal evergreen forest | BNE; BINE | Boreal evergreen forest |
| Boreal deciduous forest | BNS | Boreal deciduous forest |
| Mixed forest | N/A | Mixed forest; dense shrubland |
| Herbaceous vegetation | C3, C4 | Savanna; grassland/steppe; open shrubland; tundra |
| Desert | N/A | Desert; polar desert; rock; ice |
| No data | No data | No data |

**References**

1 Roeckner, E. *et al.* Sensitivity of simulated climate to horizontal and vertical resolution in the ECHAM5 atmosphere model. **19**, 3771-3791 (2006).

2 Zhang, K. *et al.* The global aerosol-climate model ECHAM-HAM, version 2: sensitivity to improvements in process representations. **12**, 8911-8949 (2012).

3 Vignati, E., Wilson, J. & Stier, P. M7: An efficient size‐resolved aerosol microphysics module for large‐scale aerosol transport models. **109**, doi: 10.1029/2003JD004485 (2004).

4 Ramankutty, N. & Foley, J. in *ISLSCP Initiative II Collection. Data set. Available on-line [http://daac.ornl.gov/] from Oak Ridge National Laboratory Distributed Active Archive Center* (eds F. G. Hall *et al.*) (Oak Ridge, Tennessee, U.S.A, 2010).

5 Li, J. & Zeng, Q. A unified monsoon index. **29** 1274, doi:1210.1029/2001GL013874 (2002).
